# Exploring Physicians' Willingness to Integrate Artificial Intelligence in Clinical Practice: Ethical and Practical Insights From a Jordanian Cross‐Sectional Survey

**DOI:** 10.1002/hsr2.71994

**Published:** 2026-03-04

**Authors:** Rana K. Abu‐Farha, Karem H. Alzoubi, Ala'a Al Safadi, Mervat M. Alsous, Aya Nawasreh, Maryam K. El‐zubi, Fahmi Y. Al‐Ashwal

**Affiliations:** ^1^ Clinical Pharmacy and Therapeutics Department, Faculty of Pharmacy Applied Science Private University Amman Jordan; ^2^ Department of Pharmaceutical Sciences, College of Pharmacy, QU Health Qatar University Doha Qatar; ^3^ Department of Clinical Pharmacy and Biopharmaceutics, Faculty of Pharmacy The University of Jordan Amman Jordan; ^4^ Department of Clinical Pharmacy, Faculty of Pharmacy Jordan University of Science and Technology Irbid Jordan; ^5^ Department of Pharmacy The International Medical Company Doha Qatar; ^6^ Department of Clinical Pharmacy and Pharmacy Practice, Faculty of Pharmacy University of Science and Technology Sana'a Yemen; ^7^ Department of Clinical Pharmacy, College of Pharmacy Al‐Ayen Iraqi University Thi‐Qar Iraq

**Keywords:** artificial intelligence, clinical practice, ethics, Jordan, physicians

## Abstract

**Background and Aims:**

This study explored the practical perspectives of healthcare professionals in Jordan regarding integrating artificial intelligence (AI) tools into clinical practice and describes their concerns about AI's ethical implications.

**Methods:**

The study utilized a cross‐sectional, questionnaire‐based survey that was conducted with employed physicians in Jordan from April through September 2025. The survey used a validated instrument to assess the participants' AI experience, willingness to adopt AI, practical and ethical concerns associated with AI, and support for the recommended actions. The data were analyzed using descriptive statistics and logistic regression.

**Results:**

In this study, 297 physicians participated (median age = 36.0; IQR = 19.0). Around 72% of the participants (*n* = 214) reported having prior experience with AI, while 50.8% (*n* = 151) expressed an openness to using AI tools in their clinical practice. Physician concerns about AI included a lack of ability to manage complex cases (*n* = 216, 72.8%), jeopardizing the physician–patient relationship (*n* = 204, 68.7%), and diminishing their cognitive ability (*n* = 210, 70.7%). Other ethical concerns included cultural differences (*n* = 213, 71.7%), and unclear accountabilities for any errors resulting from using AI (*n* = 209, 70.4%). Physicians who reported being more willing to adopt AI tools had significantly shorter median ages (adjusted odds ratio [AOR] = 0.971, *p* = 03) and had prior experience with AI (AOR = 0.262, *p* < 001) and had daily patient case loads of at least 10 patients (AOR = 1.895, *p* = 05).

**Conclusion:**

While Jordanian physicians recognize AI's benefits, they express significant ethical, practical, and contextual concerns. This study highlights the unique concerns of Jordanian physicians, which differ from those in other countries, and underscores the need for region‐specific policies addressing training, cultural adaptation, and regulation to support AI integration in clinical practice.

## 1 Introduction

Artificial Intelligence (AI) refers to the development of robots or computer software capable of imitating human cognition, that is, possessing the ability to process information and learn as humans do [1, 2]. These systems are designed to operate in digital and physical environments. AI has progressed rapidly in recent years; therefore, it has been adopted into a variety of industries, including drug development, healthcare, and pharmacokinetics [3, 4, 5, 6, 7]. AI is already being successfully used in clinical settings: examples include the analysis of radiological images, pathology, ophthalmology, and diabetic retinopathy [8].

The integration of AI into the clinical environment presents benefits and challenges, as well as ethical and legal issues [1]. AI can help streamline administrative processes and create customized treatment plans for patients. AI systems can also detect patterns and correlations within large amounts of medical data that human beings may not identify; therefore, AI provides more timely and accurate interventions [1, 2]. Additionally, AI can help find more cost‐effective solutions to healthcare issues and generate more rapid and accurate diagnoses.

On the other hand, there are many issues associated with the implementation of AI within the clinical arena, including accountability, integrity of the system, and the need for legal and ethical consideration around patient privacy and data security. There remains a need to develop a delicate balance between supporting the advancement of technological innovation and ensuring that technology conforms to its reasonable use in the clinical environment [9].

Physicians have varying views on how AI may impact therapeutic settings. Benefits include more precise treatment decisions and reduced time commitment for medical practitioners. However, challenges arise, such as when AI tools select a diagnosis that differs from the physician's medical diagnosis. This discrepancy may result from the technical limitations of AI [10].

Many general practitioners in the United Kingdom believe that AI will soon replace a large portion of their profession, despite their doubts about its use [11]. However, it is still unclear how AI‐provided information will impact the doctor‐patient interaction in actual practice [12].

Physicians need to be ready to lead this transformation and accept the changes AI will bring to their profession. Physicians should have extensive knowledge and expertise. For the successful adoption and implementation of technology to gain a competitive advantage, it is therefore crucial to understand how doctors generally view the use of AI in clinical care to develop legislative policies, update the information technology infrastructure, and establish and modify data privacy, data confidentiality, and code of ethics. Thus, this study aims to determine the practical perspectives of physicians and the ethical challenges of integrating AI in clinical practice in Jordan.

## 2 Methods

### 2.1 Study Design and Setting

This was a cross‐sectional, questionnaire‐based study conducted to investigate physicians' ethical and practical perspectives on the integration of AI tools, including but not limited to generative AI, into clinical practice. The study was conducted across multiple healthcare settings in Jordan, including governmental, military, and private sectors. AI was defined in this context as algorithmic technologies capable of producing textual, diagnostic, or decision‐support outputs based on complex data inputs, encompassing large language models, decision‐support systems, and other AI applications used in clinical environments.

### 2.2 Study Population and Recruitment

The study targeted any physician registered with the Jordanian Medical Association. Any physician working for either a government facility (or military) or a private hospital/clinic located within a Jordanian facility was eligible to participate in the research project. Recruitment for research subjects occurred through social media channels such as Facebook, WhatsApp, Instagram, and LinkedIn targeting professional groups/networks. Participation was voluntary with no financial or material incentives offered to participants. Surveys were conducted over a 5‐month period (April 17, 2025 through September 10, 2026).

### 2.3 Instrument Development and Content

To construct our survey instrument, we examined a number of recent studies in the literature on the application of AI in the health care field, with a focus on peer‐reviewed studies that discuss ethical and practical barriers to using AI in clinical settings. We included some of the items that have been validated from a previous survey instrument designed by Alsaedi et al., although some of the items were modified from the original version [13]. Several PhD‐level researchers (all pharmacist researchers who conduct practice‐based or pharmaceutical research) evaluated the questions on clarity, relevance, and timeliness after developing the first draft of the survey tool. The final version of the survey had five sections, with Section 5 (demographics and job history; seven closed‐ended and two open‐ended questions) and Section 2 (physicians' knowledge about and experience with AI applications; five closed‐ended questions). Section 3 (10 items related to practical issues associated with using AI in medicine), Section 4 (seven items describing the ethical issues associated with AI, such as privacy, consent, accountability, and bias), and Section 5 (six items requesting suggestions for improving AI in medicine). Section 3 was assessed using a five‐point Likert scale to measure responses to the questions from “strongly disagree” (1) to “strongly agree” (5).

Following the development of the instrument, the researcher conducted a pilot study with 40 physician respondents in order to ensure the clarity of questions and appropriateness of the questions for the target audience, as well as to determine whether or not there was uniformity in response between physicians. Internal consistency was measured using Cronbach's alpha, and the results support acceptable internal consistency for the sections of the instrument (0.846; Section 3, 0.819; Section 4, 0.871; Section 5).

### 2.4 Data Collection Procedure

Data were obtained via the use of Google Forms. The survey link was sent out on various social media channels (Facebook, WhatsApp, Instagram, and LinkedIn) aimed at Jordanian physicians. The questionnaire was self‐administered and took about 10 min to complete. No identifiable personal information was collected, and all data were saved on secure and password‐protected digital servers only accessible to the research team. Anonymity and confidentiality were maintained throughout the study, and all data will be used solely for academic research purposes.

### 2.5 Sample Size Determination

The sample size was calculated to be able to perform multivariable logistic regression analyses to identify the factors that influence physicians' willingness to use AI in their practice (dependent variable). Using the rule of thumb for logistic regression established by Peduzzi et al. which specifies that there must be at least 10 events for each predictor variable [14] and there are approximately 13 independent predictor variables in this analysis, a minimum of 130 physicians who have expressed a willingness to use AI must be included in order to have sufficient statistical power at an alpha of 0.05 and a power of 80% for the analysis.

### 2.6 Ethical Considerations

The study was reviewed and approved by the Institutional Review Board of the Applied Science Private University (IRB Approval No. 2025‐PHA‐26) and was performed in accordance with the ethical principles outlined in the Declaration of Helsinki. The online questionnaire contained a link to an electronic consent form at the top of the questionnaire. The consent form provided a description of the study, the procedures to participate in the study, risks, benefits, voluntary nature of participation, and how confidentiality of the data would be maintained. All respondents had to indicate their consent before proceeding with completing the online survey by selecting the “yes” option.

### 2.7 Statistical Analysis

Once the data collection process had finished, researchers reviewed each response for completeness and accuracy. The data were entered into and analyzed using the Statistical Package for Social Sciences (SPSS) version 22. (SPSS Inc., Chicago, IL, USA). Researchers used descriptive statistics to summarize participant characteristics and presented categorical variables as frequency counts and percentages, and continuous variables as median values and interquartile ranges (IQR).

To identify independent predictors influencing physicians' willingness to adopt AI in the clinical setting, the researchers used logistic regression analysis and followed a two‐step procedure. First, the researchers ran separate univariate logistic regression analyses on each of the predictor variables. The predictor variables that had a *p*‐value of < 25 in the univariate analysis were selected for inclusion in the multivariable logistic regression model. For purposes of this analysis, “No” and “Unsure” responses regarding willingness to adopt AI were combined into a single response category to streamline the interpretation of these results. The researchers assessed the model fit using the −2 Log Likelihood value and pseudo‐*R*² statistics (Cox & Snell *R*² and Nagelkerke *R*²), and evaluated whether multicollinearity existed in the predictor variables using the variance inflation factor. A *p*‐value of < 05 was considered statistically significant for all analyses.

## 3 Results

This study included 297 physicians, with a median age of 36.0 years (IQR = 19.0) and a median of 6.0 years of clinical experience (IQR = 10.0). The majority of participants were male (*n* = 211, 71.0%). The most frequently reported specialties were surgery, internal medicine, and pediatrics, while fewer participants specialized in psychiatry, radiology, and ophthalmology. Most respondents obtained their medical degrees from Jordanian universities (*n* = 240, 80.8%).

Physicians were mainly employed in governmental (*n* = 129, 43.4%) and private (*n* = 109, 36.7%) healthcare institutions, with a smaller proportion working in the military sector (*n* = 59, 19.9%). The majority practiced in urban areas (*n* = 209, 70.4%). Daily patient load varied across the sample, with roughly one‐third of physicians seeing fewer than 10 patients per day (*n* = 94, 31.6%), one‐third seeing 10–20 patients (*n* = 104, 36.7%), and the remaining third seeing more than 20 (*n* = 94, 31.6%). Additional details on the participants' sociodemographic and professional characteristics are presented in Table 1.

**Table 1:** Sociodemographic and professional characteristics of the participating physicians (*n* = 297).

Parameter	Median (IQR)	Frequency (%)
Age	36.0 (19.0)	
Gender		
Male		211 (71.0)
Female		86 (29.0)
Education level		
Bachelor of Medicine		257 (86.5)
Graduate degree (MSC or PhD)		40 (13.5)
Years of experience	6.0 (10.0)	
Specialty		
General practitioner		29 (9.8)
Internal medicine		60 (20.2)
Pediatrics		41 (13.8)
Surgery		92 (31.0)
Obstetrics and gynecology		17 (5.7)
Psychiatry		6 (2.0)
Dermatology		17 (5.7)
Radiology		1 (0.3)
Emergency medicine		9 (3.0)
Anesthesiology		13 (4.4)
Family medicine		10 (3.4)
Ophthalmology		2 (0.7)
Country of graduation		
Jordan		240 (80.8)
Others		57 (19.2)
Type of healthcare institution		
Private sector		109 (36.7)
Military sector		59 (19.9)
Governmental sector		129 (43.4)
Location of healthcare institution		
Urban area		209 (70.4)
Rural area		88 (29.6)
Number of patients seen daily		
< 10		94 (31.6)
10–20		104 (36.7)
> 20		94 (31.6)

Abbreviation: IQR, interquartile range

As shown in Table 2, the majority of participants (*n* = 214, 72.1%) reported having prior experience with AI, while 27.9% did not (*n* = 83). In addition, more than half of them indicated willingness to use AI tools in their clinical work (*n* = 151, 50.8%), whereas a smaller proportion were either unsure (*n* = 101, 34.0%) or not willing (*n* = 45, 15.2%).

**Table 2:** Physicians’ experience and awareness regarding AI in clinical practice (*n* = 297).

Parameter	Frequency (%)
Previous use of any AI tool or technology in clinical practice	
Yes	214 (72.1)
No	83 (27.9)
Willingness to use AI tools	
Yes	151 (50.8)
No	45 (15.2)
I don't know	101 (34.0)
Awareness of potential benefits of AI	
Yes	241 (81.1)
No	56 (18.9)
Awareness of potential concerns of AI	
Yes	194 (65.3)
No	103 (34.7)
Awareness of AI tool availability in hospitals in Jordan	
Yes	134 (45.1)
No	163 (54.9)

The vast majority of respondents were aware of the potential benefits of AI (*n* = 241, 81.1%), and most also reported awareness of potential concerns (*n* = 194, 65.3%). However, fewer than half were aware of the availability of AI tools in hospitals across Jordan (*n* = 134, 45.1%).

Regarding physicians' concerns about the integration of AI in their clinical practice, the data show several key findings (Table 3). Most physicians (*n* = 216, 72.8%) expressed concern that AI might not understand complex medical conditions as accurately as they do. Similarly, 72.8% (*n* = 216) agreed/strongly agreed that AI could worsen healthcare issues such as overutilization, overdiagnosis, and overtreatment. Over two‐thirds of respondents worried that AI might weaken the relationship between patients and their treating physicians (68.7%, *n* = 204) and that not all physicians have adequate skills to use AI effectively (*n* = 211, 71.1%). Furthermore, 70.7% (*n* = 210) believed that AI could negatively impact physicians' analytical thinking, critical thinking, and decision‐making skills.

**Table 3:** Physicians’ concerns about the integration of AI in clinical practice (*n* = 297).

Statement	Strongly agreed/agreed (%)	Neutral (%)	Strongly disagreed/disagreed (%)
AI might not understand complex medical conditions as accurately as physicians do.	216 (72.8)	48 (16.2)	33 (11.1)
AI could reduce the roles that physicians traditionally play.	196 (66.0)	60 (20.2)	41 (13.8)
Physicians might feel more stressed because of the additional demands of using technology.	177 (59.6)	79 (26.6)	41 (13.8)
AI could potentially weaken the relationship between patients and their treating physicians.	204 (68.7)	57 (19.2)	36 (12.1)
Not all physicians have adequate skills to use AI effectively	211 (71.1)	65 (21.9)	21 (7.0)
There is a concern that AI‐based system could be manipulated from outside (terrorists, hackers … etc.)	192 (64.6)	89 (30.0)	16 (5.4)
AI will worsen problems in healthcare, such as overutilization of laboratory testing, overdiagnosis, and overtreatment.	216 (72.8)	87 (29.3)	16 (5.4)
The use of AI may negatively impact physicians’ analytical thinking, critical thinking, and decision‐making skills.	210 (70.7)	57 (19.2)	30 (10.1)
AI lacks contextual knowledge and the ability to read social cues.	196 (66.0)	73 (24.6)	28 (9.4)
Physicians lack the time to learn how to use complex AI‐based medical devices.	183 (61.6)	80 (26.9)	34 (11.5)

Regarding physician concerns about ethical challenges in integrating AI in clinical practice (Table 4), the most prominent issues included cultural sensitivity (*n* = 213, 71.7%), where AI algorithms developed in one cultural context may not be effective across diverse populations; accountability and responsibility for AI‐related medical errors (*n* = 209, 70.4%), highlighting uncertainty over who is liable when AI causes harm; data ownership and control (*n* = 205, 69.1%), concerning the ethical and legal sharing or selling of medical data used for AI training; and patient privacy and data security (*n* = 204, 68.7%), reflecting fears that sensitive data might be inadequately protected. Other major issues included ethical dilemmas between borders (*n* = 203, 68.4%), as well as the difficulty of obtaining valid informed consent (*n* = 188, 63.3%) because doctors may not be able to properly explain AI's functionality to a patient, etc. Equitable access for patients (*n* = 173, 58.2%) highlighted the concern of possible bias within AI technologies, creating inequitable health care access.

**Table 4:** Ethical challenges of integrating AI in clinical practice as perceived by physicians (*n* = 297).

Statement	Strongly agreed/agreed (%)	Neutral (%)	Strongly disagreed/disagreed (%)
Security and safety: Patient privacy and data security may be inadequately addressed in the integration of AI systems in hospital practices.	204 (68.7)	67 (22.6)	26 (8.8)
Patient equity: Bias in AI tools may result in unfair healthcare delivery.	173 (58.2)	91 (30.6)	33 (11.1)
Informed consent: Ensuring appropriate informed consent becomes challenging when medical professionals are unable to effectively explain the functioning of AI medical devices to patients.	188 (63.3)	97 (32.7)	12 (4.0)
Accountability and responsibility: There is a concern about who is responsible if AI makes medical errors without healthcare professionals’ input.	209 (70.4)	74 (24.9)	14 (4.7)
Data ownership and control: Determining who owns the medical data used to train AI systems and how it can be ethically and legally shared or sold.	205 (69.1)	80 (26.9)	12 (4.0)
Cross‐border Issues: AI in healthcare often involves international collaborations and data sharing, which raises ethical challenges concerning regulatory differences and standards used for patient care.	203 (68.4)	74 (24.9)	20 (6.7)
Cultural sensitivity: AI algorithms developed in one culture may not be appropriate or effective when applied to diverse populations with different cultural norms.	213 (71.7)	67 (22.6)	17 (5.7)

**Figure 1:**
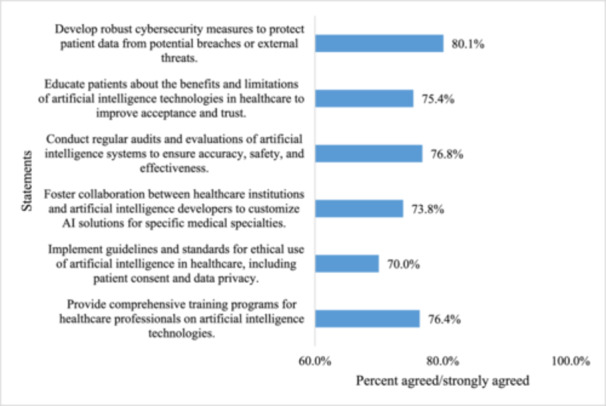
Physician‐endorsed recommendations to facilitate AI integration in clinical practice (*n* = 297).

The majority of physicians surveyed agreed with many important recommendations regarding the use of AI in clinical practice (Figure 1). The top three recommendations that received the most support were developing comprehensive cybersecurity protocols (*n* = 238; 80.1%), performing ongoing audits of all AI systems (*n* = 228; 76.8%), and providing AI training for health care workers (*n* = 227; 76.4%). Other recommendations that received a significant amount of support included educating patients about the use of AI, encouraging collaborative work between health care providers and the developers of AI systems, and establishing ethical guidelines.

Following the conduction of the simple and multiple logistic regression analysis (Table 5), several factors were significantly associated with physicians' willingness to adopt AI tools in clinical practice. Younger age was associated with greater willingness to adopt AI (adjusted odds ratio [AOR] = 0.971, *p* = 0.03). Physicians who had previously used AI tools were significantly more likely to express willingness to adopt them (AOR = 0.262, *p* < 0.001). Furthermore, Physicians who saw 10 or more patients per day were nearly 1.9 times more likely to be willing to adopt AI tools in clinical practice compared to those seeing fewer than 10 patients daily (AOR = 1.895, *p* = 0.05).

**Table 5:** Factors associated with physicians’ willingness to adopt AI tools in clinical practice (*n* = 297).

Predictors	Dependent variable: Willingness to adopt AI tools in clinical practice [0: No/I don't know, 1: Yes]					
	COR	95% CI of the COR	*p*‐value^#^	AOR	95% CI of the AOR	*p*‐value^$^
Age (years)	0.950	0.930–0.969	< 0.001^^^	0.971	0.947–0.997	0.03*
Gender						
Male	Reference					
Female	1.018	0.617–1.682	0.94	—		—
Education level						
Bachelor of Medicine	Reference					
Graduate degree (MSC or PhD)	0.083	0.029–0.240	< 0.001^^^	0.291	0.079–1.067	0.06
Years of experience	0.993	0.971–1.014	0.49	—		—
Specialty						
General practitioners	Reference					
Specialist	0.188	0.070–0.506	0.001^^^	0.371	0.123–1.125	0.08
Country of graduation						
Jordan	Reference					
Others	3.028	1.611–5.692	0.001^^^	1.476	0.700–3.114	0.31
Type of healthcare institution						
Governmental/military sectors	Reference					
Private Sector	0.544	0.337–0.877	0.01^^^	0.865	0.475–1.575	0.64
Location of healthcare institution						
Urban area	Reference					
Rural area	0.495	0.298–0.823	0.007^^^	0.952	0.482–1.879	0.89
Number of patients seen daily						
< 10	Reference					
≥ 10	2.745	1.648–4.571	< 0.001^^^	1.895	1.007–3.565	0.05*
Prior experience with AI tools						
Yes	Reference					
No	0.140	0.076–0.258	< 0.001^^^	0.262	0.124–0.556	< 0.001*
Awareness of potential benefits of AI						
Yes	Reference					
No	0.314	0.167–0.592	< 0.001^	0.533	0.221–1.286	0.16
Awareness of potential concerns of AI						
Yes	Reference					
No	0.186	0.109–0.318	< 0.001^	0.563	0.245–1.291	0.18
Awareness of AI tool availability in hospitals in Jordan						
Yes	Reference					
No	0.492	0.309–0.784	0.003^	1.115	0.586–2.122	0.74

*Note:* ^#^Using simple logistic regression. ^$^Using multiple logistic regression. ^^^Eligible for entry in multiple logistic regression. *Significant at 0.05 significance level. Abbreviations: AOR, adjusted odds ratio; COR, crude odds ratio.

The multiple logistic regression model was statistically significant and explained a moderate proportion of the variance in physicians' willingness to adopt AI tools in clinical practice. The model accounted for approximately 27.6% of the variance according to the Cox & Snell *R*² and 36.8% according to the Nagelkerke *R*². The final model had a –2 Log Likelihood value of 315.827, indicating a reasonable model fit.

## 4 Discussion

This initial evidence indicates the readiness of Jordanian physicians to adopt AI within their practice. While almost half of all surveyed practitioners expressed enthusiasm in adapting AI to their practices, the remaining physicians demonstrated reluctance and skepticism. Cautious optimism is indicative of physicians recognizing AI to be potentially beneficial, while continuing to be concerned about the safety, accountability, and appropriateness of it for their patients. A comparison of our findings with the literature indicates that Jordanian physicians face similar challenges to healthcare workers in other parts of the world, but also that they have additional challenges. Notably, our study reveals a novel disconnect between physicians' awareness of AI and their access to it, as well as concerns about the cultural relevance of AI technologies in the Jordanian healthcare context. These unique challenges stem from Jordan's specific infrastructure limitations, cultural context, and regulatory environment.

Younger physicians and physicians who have engaged with AI previously show higher levels of willingness to incorporate AI into their practice; this pattern has also been confirmed in the literature throughout the world. For example, younger practitioners and medical students who are digitally literate were shown to be more receptive to adopting AI [15], while the lack of practical experience with AI was cited as a barrier to willingness to use AI by Jeddah‐based practitioners [16]. The inconsistency between younger and more senior practitioners highlights the necessity for AI training to incorporate an understanding of age‐related differences that will provide an equitable opportunity for all healthcare workers to adopt AI [15, 16].

Physicians surveyed in this study showed concerns about AI's limitations in understanding complicated cases, the possibility of overdiagnosing patients, the concern for data privacy, and the possibility that using AI may harm the relationship between doctors and patients. Many of these same worries were present in other regional countries. For example, physicians in Bahrain recognized that AI can improve efficiency; however, they are concerned about losing their jobs and developing insufficient skills [17]. Similarly, physicians in Saudi Arabia expressed concern about a lack of autonomy, unclear consent, and legal liability caused by using AI [13, 18]. The general ambivalence about utilizing AI rests on a worldwide scale. General practitioners from the United Kingdom are concerned that AI will cause them to be less able to build relationships with patients [19]. Physicians in Germany are demanding that AI systems be open and verifiable [20], while physicians in Italy report producing more efficiently but continue to distrust “black box” devices [21]. A recent review of the literature indicates that when considering the possible benefits of using AI, users frequently consider the associated risks [22, 23, 24, 25]. Unless these concerns are addressed through a set of agreed‐upon international standards, the use of AI will likely continue to be limited, especially in Jordan, where there is an opportunity to improve access to care by implementing AI.

A major insight from our investigation is the disparity between knowledge about artificial AI in Jordan and access to it, as measured by the responses from Jordanian physicians in the sample. Although 81.1% of the physicians reported knowing that AI would benefit them professionally, fewer than half of them also reported having access to AI resources/technology within their own medical facilities. This disconnect exists throughout the Middle East region, as seen in Saudi Arabia, where practitioners provided positive feedback about AI yet had almost no hands‐on experience using it [26]. Given that Jordan's infrastructure and financial resources are inadequate compared with those of more developed countries, it is likely that the gap between practitioners' awareness of AI and their ability to use it successfully would be even greater than in countries like Saudi Arabia—growing the gap in health status between the two countries unless specific investments are made.

The alignment of AI solutions with their cultural and epidemiological contexts is a concern among respondents. Approximately 70% of respondents indicated they did not know whether or not the AI technologies that have been imported from the west are culturally appropriate or viable in their home countries. Similar observations have been made in the United Arab Emirates as well [27]. Given that personal relationships and trust between patients and caregivers are critical elements of culturally competent healthcare in the Middle Eastern region, it cannot be overstated how significant cultural compatibility is in relation to AI application. If the data used to train AI applications are exclusively western in origin, then these applications will likely be biased in their outputs. This loss of credibility will likely result in the creation of health disparities for individuals and/or populations within the respective jurisdictions.

Jordanian physicians supported establishing guidelines for using AI in healthcare, including training, ethics, cybersecurity, patient education, etc. Similar sentiments were expressed from physicians from both Saudi Arabia and Europe. These situations demonstrate the global need for an implementation of AI within healthcare that is both transparent and regulated [20, 21, 28]. Ethical issues represent the most critical aspects to the successful implementation of AI, namely trust, accountability, and fairness. Weiner et al. write that successful implementation of AI will only occur if physicians have autonomy and that patients continue to have trust in physicians. In a care‐oriented patriarchal culture like Jordanian culture, hospitals may be inadvertently violating fully informed consent of patients if patients are not included in the governance of AI [12].

This research is the first of its kind that examines how medical professionals view AI, providing a major link between the vast amount of research conducted in the United Arab Emirates and Saudi Arabia and the limited amount conducted in Jordan. The research has also enhanced our comprehension about how a country/region must be prepared to implement an intelligent system. In addition to creating a better understanding of both general challenges, for example, ethical expectations and shortfalls in training, and the unique geographical challenges faced by Jordan that make integrating AI into medical practice difficult (e.g., lack of access to AI, awareness of AI).

In addition, the research reveals many serious policy‐level challenges to integrating AI into practice for health professionals (e.g., building a legal framework and standards for AI use within the medical setting; financing infrastructure necessary to develop and use AI; and engaging citizens in the decision‐making process to help develop trust in AI). If the current policy challenges can be addressed with a collaborative and strategic plan and successfully implemented, Jordan can fulfill its national vision of using AI to create a fair and efficient health care system in Jordan.

This study has its limitations, which deserve to be recognized. First, the cross‐sectional design, which measures perceptions at one specific point, does not allow for establishing causal relationships or tracking changes over time. In addition, while the sample size of 297 physicians was sufficient to allow for logistic regression based on our power calculations, it is possible that it limited the generalizability of the results because participants were obtained through social media, which may have created a selection bias towards tech‐savvy or urban physicians. Second, we were unable to ascertain the actual number of respondents who viewed the survey because it was administered online, making it impossible to calculate the real response rate. Lastly, because we relied on self‐reported data using social media platforms, we may have experienced response bias, including the “social desirability” effect, in which respondents overstate their awareness or intentions to adopt AI. Furthermore, there is a risk associated with using social media to recruit study participants, and there is a possibility that many of the study participants were not actual physicians. Importantly, there was no qualitative component to the study that could have provided insight into the nuances associated with the quantitative data, nor did the study assess actual behaviors related to AI use. Finally, by only recruiting Jordanian physicians, there are no perspectives from other stakeholders, such as patients or other healthcare managers or policymakers, that contribute to a more comprehensive understanding of the challenges by incorporating various stakeholders' views and opinions.

## 5 Future Directions

Future studies should include a more diverse group of physicians from various geographic locations and a wider age distribution to increase the generalizability of these findings. Longitudinal studies of physician attitudes toward AI technology or the adoption of AI technology over time would also be valuable. Qualitative research might also provide insight into physicians' concerns regarding AI technology implementation and how cultural characteristics influence AI uptake in medicine.

## 6 Conclusions

This study presents new insights into the perceptions of Arab physicians regarding the use of AI in the provision of healthcare in Jordan. Physicians view the potential benefits of utilizing AI in healthcare, but have expressed concerns regarding accessibility, cultural relevance, and ethical protections. The findings underscore the need for planning at both the policy level and the level of practitioners, training for physicians, and development of tools and processes that meet the needs of the Jordanian populace. If implemented appropriately, technology can be used to deliver equitable, effective care in Jordan and around the world.

## Author Contributions

**Rana K. Abu‐Farha:** conceptualization, writing – review and editing, writing – original draft, visualization, methodology, formal analysis, data curation, investigation. **Karem H. Alzoubi:** writing – original draft, writing – review and editing, conceptualization, methodology, investigation, data curation. **Ala'a Al Safadi:** writing – original draft, writing – review and editing, conceptualization, methodology. **Mervat M. Alsous:** writing – original draft, writing – review and editing, conceptualization, methodology. **Aya Nawasreh:** writing – original draft, writing – review and editing, conceptualization, methodology. **Maryam K. El‐zubi:** writing – original draft, writing – review and editing, conceptualization, methodology. **Fahmi Y. Al‐Ashwal:** writing – original draft, writing – review and editing, conceptualization, methodology.

## Funding

The authors received no specific funding for this work.

## Disclosure

All authors have read and approved the final version of the manuscript. The corresponding author, Fahmi Y. Al‐Ashwal, had full access to all of the data in this study and takes complete responsibility for the integrity of the data and the accuracy of the data analysis.

## Conflicts of Interest

The authors declare no conflicts of interest.

## Transparency Statement

The lead author Fahmi Y. Al‐Ashwal affirms that this manuscript is an honest, accurate, and transparent account of the study being reported; that no important aspects of the study have been omitted; and that any discrepancies from the study as planned (and, if relevant, registered) have been explained. The lead author (Rana K. Abu‐Farha) affirms that this manuscript is an honest, accurate, and transparent account of the study being reported; that no important aspects of the study have been omitted; and that any discrepancies from the study as planned (and, if relevant, registered) have been explained.

## Data Availability

The raw data used to support the findings of this study are available from the corresponding author, Fahmi Y. Al‐Ashwal, upon reasonable request.
